# Puerperal Eclampsia, Etc.

**Published:** 1870-01

**Authors:** O. C. Gibbs

**Affiliations:** Frewsburg, Chautauqua Co., N. Y.


### Puerperal Eclampsia, etc.

*To the Editor Buffalo Medical and Surgical Journal:*

Dear Sir:—It seems to me that our mutual friend and your frequent contributor is a little unfortunate at time in giving advice to younger members of our profession. One grain of morphine, hypodermically administered *every half hour,* he thinks not too much, so long as the convulsions last.

Under this treatment we should expect a *complete suspension* of the convulsions in from one and a half to two hours, *as well as the suspension of the life of the patient!*

I believe it is admitted by all, that medicines hypodermically, are more speedily and more powerfully operative, even in the same dose, than when administered by the mouth—the reason being the whole of the dose must, of necessity, enter the circulation at once—whereas, when administered by the mouth, it must commingle with the contents of the stomach, whether great or little, and consequently enters the system slowly and gradually.

That the dose referred to may be judiciously administered, and even repeated once, *under certain circumstances,* I am not going to deny. (Though I may here be allowed to express my doubt if such a dose, by actual weight, has ever been fully and perfectly administered hypodermically, and repeated in half an hour, and the *patient survived!*)

I simply wish to say that I regard this statement in your July Number as a suitable text and occasion for giving my own views upon the use of opium in puerperal convulsions.

I have not the time or inclination to write, or you the space to publish, or your subscribers the patience to read, a full expression of my views upon the pathology of puerperal eclampsia.

But I do hope to give a few practical hints that may be of benefit to the inexperienced, who may, unwisely, have tried this plan of treatment and failed, that they may possibly conjecture *why* they have failed.

The indications for the use of opium in convulsions, or in any other disease, where the brain plays an important part, are plain and umistakable. *If the pupil of the eye is contracted much beyond its normal condition, opium is always, and under all circumstances, in any dose, a positive injury. If the pupil is enlarged beyond its normal condition, opium may be administered (allowing time for its action), in any dose that will not reduce the pupil much below the normal condition!*

This is the point I wish to make, and the rule I wish to lay down for the guidance of the inexperienced. To give opium in a certain disease, in certain doses, regardless of conditions, manifests ignorance and want of discrimination.

In puerperal convulsions, of an hysterical type, there is no remedy that can begin to compare with chloroform—it will always control them. But in the apoplectic form of puerperal convulsions what can we say of it? The same that we can of one grain doses of morphine—it will invariably kill the patient!

It is only in the hysterical and epileptical form of puerperal convulsions, that either opium or chloroform can be administered with benefit, or even safety. In either of the two, opium may be administered with benefit, but not in doses of one grain sulphate of morphine every half hour, *ad libitum*, hypodermically administered!

In the apoplectic form of puerperal convulsions, whoever administers morphine in one grain doses hypodermically, will in our opinion have no reason to boast of his success.

Yours truly, O. C. Gibbs, M. D.,

Frewsburg, Chautauqua Co., N. Y.

